# Quaternary fluvial terraces of the Tiber Valley: geochronologic and geometric constraints on the back-arc magmatism-related uplift in central Italy

**DOI:** 10.1038/s41598-017-02437-1

**Published:** 2017-05-31

**Authors:** Fabrizio Marra, Fabio Florindo, Carmelo Petronio

**Affiliations:** 1 Istituto Nazionale di Geofisica e Vulcanologia, Via di Vigna Murata, 605 – 00143 Rome, Italy; 2 Dipartimento di Scienze della Terra, Sapienza, Università di Roma, P.le Aldo Moro 5, 00185 Rome, Italy

## Abstract

Through a geomorphological study relying on statistically assessed classes of hilltop elevations, we reconstruct a suite of paleo-surfaces along the Tiber River Valley north of Rome that we identify as fluvial terraces formed by interplay between global sea-level fluctuations and regional upift. Using biostratigraphic constraints provided by marine through continental deposits of Santernian age, we recognize the oldest terrace in this area, corresponding to an early coastal plain of late Santernian-Emilian age. By assuming the simple chronological principle of a staircase geometry we correlate the sea-level highstands of MIS 21 through MIS 5 with the lowest eight paleo-surfaces. By plotting against time the cumulated terrace elevations and the average elevation of the Santernian coastline in the investigated area, we detect rates of uplift during the last 1.8 Ma. Two major pulses of uplift are recognized 0.86 through 0.5 Ma, and 0.25 Ma through the Present, which are interpreted as driven by the subduction process and uprising of metasomatized magma bodies on the Tyrrhenian Sea Margin of central Italy, superimposied on a smaller isostatic component of uplift.

## Introduction

**Figure 1:**
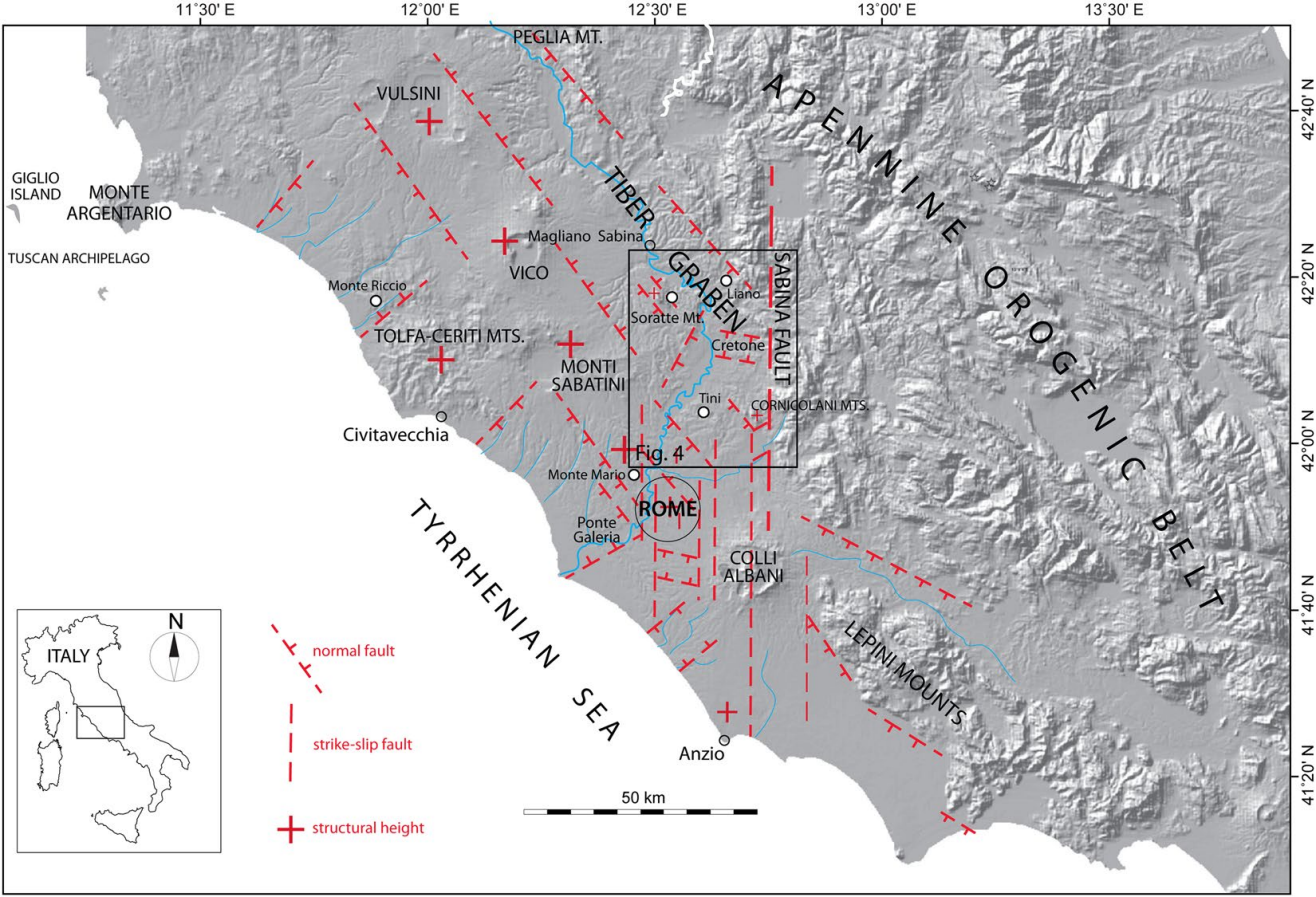
Location map. Digital Elevation Model (DEM) image showing the structural setting of the area on the Tyrrhenian Sea Margin of Latium subjected to continental sedimentation since the late Santernian, and the location of the sites providing biostratigraphic constraints to the Santernian-Emilian sedimentary basins. Modified after TINITALY/01 square WA 6570, used with permission of the Istituto Nazionale di Geofisica e Vulcanologia, Rome.

**Figure 2:**
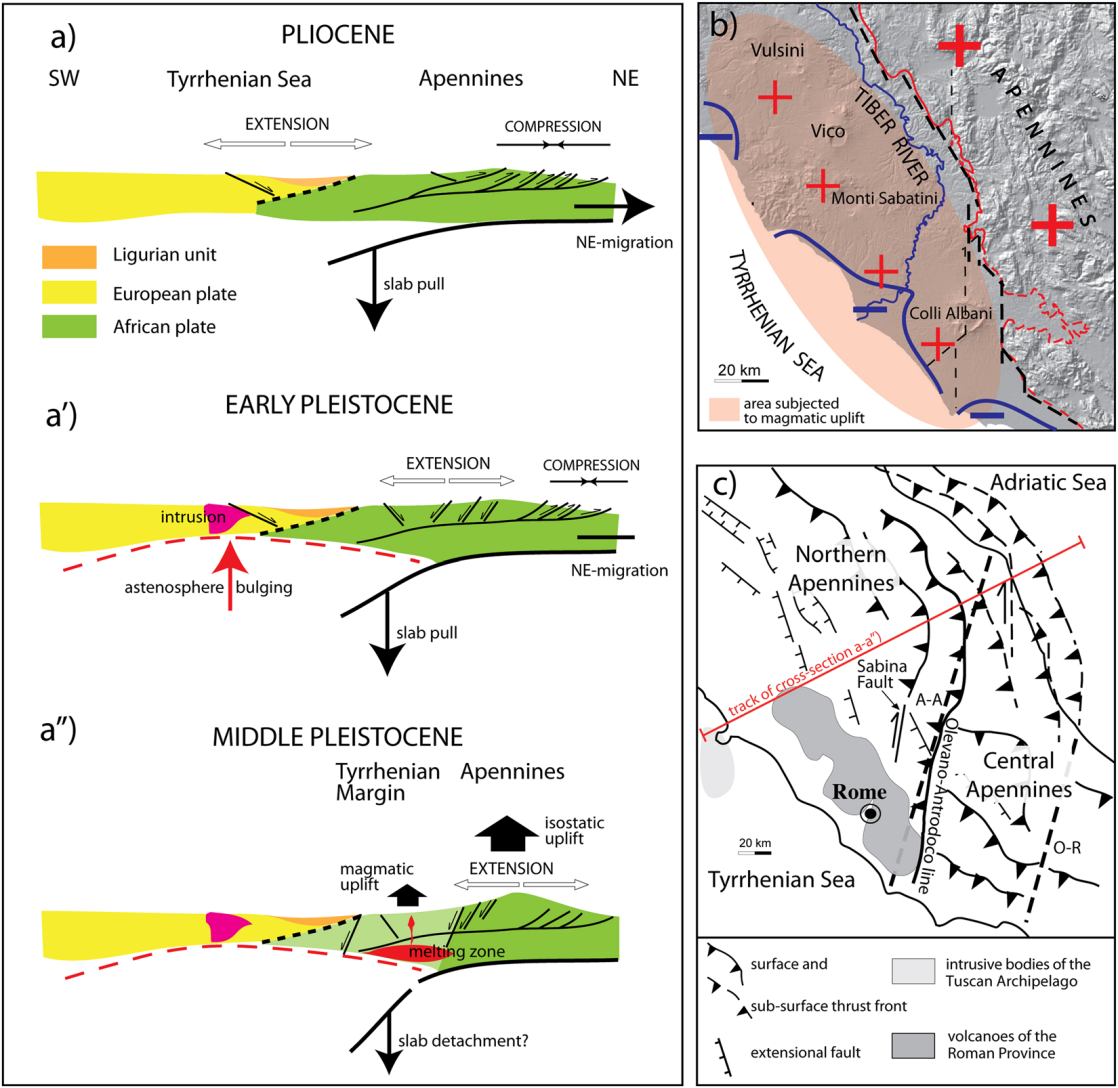
Cross-section showing the geodynamic elements responsible for the evolution of the Tyrrhenian Sea margin of Italy^3^ during Pliocene (**a′**) and Pleistocene (**b**–**b′**) times. (**b**) Map view showing the different components of the regional uplift; DEM modified after TINITALY/01 square WA 6570, used with permission of the Istituto Nazionale di Geofisica e Vulcanologia, Rome. (**c**) Structural scheme of central Italy; map hand drawn with Adobe© Illustrator CS3 13.0.0 graphic program. See text for comments.

The Tyrrhenian Sea Margin of Latium (Fig. 1), throughout the Pliocene and part of the Early Pleistocene, hosted the marine sedimentary basins produced by extensional tectonics acting at the back of the Apennines orogenic belt^1, 2^. The opening of the Tyrrhenian Sea was the consequence of the retreat of a subducting slab and the NE migration of an arched thrust and fold belt (Fig. 2a), originated by convergent African and Euro-Asian plate movement. The origin and progressive NE migration of the northern Tyrrhenian Sea basins since early Oligocene times was controlled by a delamination process causing crustal thinning and astenosphere bulging, which culminated in the emplacement of intrusive granitic bodies at the center of the back-arc region, corresponding to modern Tuscan archipelago^3^ (Fig. 2a′). Continuous NE migration of the post-orogenic extensional domain caused the shift of the collapsing sector towards the mountain range, which was dislocated by principal NW-SE trending normal faults, bordering the incoming sedimentary basins. This migration was accompanied by parallel shifting of the volcano-tectonic processes related to uprising of magma through the crust, leading to an early, acid Pliocene volcanism^4, 5^, and culminating in the Middle Pleistocene high potassic volcanism of the Roman Province^6^ (Fig. 2a″). Regional uplift, linked with the subduction process and the upwelling of methasomatized magma bodies^7, 8^, caused the progressive emersion of this area since the end of the Santernian, around 1.5 Ma, leading to widespread continentalization since ~1 ma^5^. The birth of a NW-SE chain of high-potassic volcanic districts (Roman Magmatic Region, including Vulsini, Vico, Monti Sabatini, Colli Albani; ref. 6) has strongly influenced the paleogeography of this sector since 0.8 Ma. The Tyrrhenian margin was characterized by an inland NW-SE oriented tectonic depression (“Paglia-Tiber graben”, refs 9 and 10; Tiber Graben in Fig. 1), hosting the course of the Paleo-Tiber River, and by an outer coastal area, where the large Paleo-Tiber delta and a series of minor alluvial coastal plains are located, at the mouth of smaller water courses draining the southwestern flanks of the volcanic region (Fig. 1).

A major N-S crustal discontinuity (Sabina Fault) crossing this region in correspondence of the Colli Albani volcanic district causes a sharp bend of the Tiber valley, and a southward step of the Apennines in correspondence of the Lepini Mounts (Fig. 1). This crustal discontinuity also controlled the position of the Paleo-Tiber delta, which was located between Rome and the Ponte Galeria area, and was progressively shifted to the southwest by three major phases of uplift affecting the Tyrrhenian Sea Margin around 800 ka and 600 ka, and since 200 ka^11, 12^. The northwestern boundary of the coastal area affected by the most recent uplift is represented by the Monte Argentario promontory; to the southwest, the uplift was confined by the Anzio structural height (Figs 1 and 2a). Decoupling between the vertical movements in the Apennine region and the Tyrrhenian margin, released along the border faults of the Apennine range, is interpreted as due to two different main sources linked with the geodynamics of the orogenic belt and the back-arc region (Fig. 2b). The uplift in the wider Apennines area is driven by isostatic adjustment triggered by the thickened crustal wedge (ref. 13, and references therein). In contrast, astenospheric bulging and uprising of magma from the lower crust (ref. 3, and references therein) overprinted regional isostasy and minor subsidence in the delta of the Tiber River in the Tyrrhenian margin (red shaded area in Fig. 2a) during Middle Pleistocene. This concomitant tectonic movements with different magnitude and direction are represented in Fig. 2a by the symbols + and −.

As a result of this overall uplifting regime, the continental, fluvial-lacustrine and coastal deposits in this area formed a widespread pattern of terraces that, similar to other regions in the world (e.g.: ref. 14), are organized in a staircase geometry, with the oldest surfaces at highest elevation. In the present work we have adopted the same methodological approach employed by^12^ in the coastal area between Civitavecchia and Anzio and in the inland area of Rome, to reconstruct a suite of paleo-surfaces occurring at different elevation along the inner portion of the Tyrrhenian Sea margin comprised between Magliano Sabina and Rome (Fig. 1), and to correlate them with the marine isotopic stage (MIS) timescale, providing an indirect age for each terrace.

Moreover, we review biostratigraphic data from four sections (Liano, Tini, Monte Riccio, Monte Mario) in which Santernian marine and transitional deposit occur, providing age constraint on the regressive phase leading to the continentalization of this area.

Results of this study provide insights into the uplift rates in this region and into the geodynamic processes, including volcanism and tectonics, which controlled its evolution.

### Previous studies in the investigated area

A paleo-shoreline with decreasing altitude from ca. 480 m above sea level (a.s.l.) in the NW area to ca. 220 to the SE, consistent with a differential uplift due to the development of Plio-Pleistocene magmatism in central Italy^4, 5^, was reconstructed by^10^ between the Peglia and the Cornicolani Mountains, along the western margin of the Central Apennines (Fig. 1). A Santernian age was attributed to this paleo-shoreline based on measurements of ^87^Sr/^86^Sr ratios in corals and mollusk shells collected from the nearshore deposits^10, 15^. The Santernian age of the paleo-shoreline was questioned by^16^, mainly on the basis of the supposed Middle Villafranchian age of a series of continental deposits cropping out in the vicinity of this paleoshoreline, around 270 m a.s.l., in the Sabina region (Bocchignano, Castel San Pietro, Torre Baccelli, Stazzano), which according to the authors evidenced a Gelasian age for it. However, the age revision was rejected by^17^, who remarked on the accuracy of their dating method, and pointed out the contradictory paleontological evidence at the Bocchignano and San Pietro localities^18, 19^.

Based on the presumed Santernian age^10^, also correlated with this paleo-shoreline are the deposits of a 3rd order sequence composed of:

i. a transgressive member including marine shelf, near-shore and deltaic sediments (Chiani-Tevere Formation, CTF; ref. 15, 2.1 to 1.5 Ma old (late Gelasian-Santernian);
ii. fluvial and lacustrine deposits filling the intramountain basins at the rear of the paleo-shoreline and laterally contiguous to the CTF;
iii. a regressive member including fluvial-lacustrine carbonate and terrigenous sediments (Giove Formation, GF; ref. 15), referred to the Emilian sub-stage (1.5–1.2 Ma).

Moreover, based on a synthesis of previous sedimentologic and geomorphologic studies conducted in the Middle Valley of the Tiber River^10, 20, 21^ recognized a staircase of four aggradational fluvial terraces of the Tiber River, flanking the Holocene alluvial plain, below the oldest deposit of the syngenetic uplift phase (Peperino Formation) forming a tabular plateau at 250–270 m a.s.l. This highest surface is dated by^10^ at 1.3 Ma, based on age of the rhyodacitic ignimbrite belonging to the Mt. Cimino Volcanic District^5^, responsible for the formation of the pyroclastic plateau. Each one of the four aggradational fluvial terraces, composed of channel-related and floodplain facies, gently and regularly decreases in elevation in a NNW–SSE direction and is covered and chronologically constrained by progressively younger volcanic products, correlated by^10^ with those erupted by the Vulsini, Vico, and Monti Sabatini districts. Based on these proposed correlations, the first fluvial terrace spans the interval 1.2 to 0.6 Ma, and dips from 330 to 170 m a.s.l. The deposits of the second terrace overly the Tufo Giallo della Via Tiberina pyroclastic-flow deposit (546 ± 3 ka, ref. 22), and is therefore dated from 0.5 to 0.3 Ma, and the related paleo-surface dips from 210 to 65 m. The third terrace is constrained to 200–150 ka by the underlying Vico Lake Lava Flows (0.3–0.25 Ma, ref. 23) and by the overlying Tufo Rosso a Scorie Nere Vicano (0.15 Ma, ref. 5); it dips from 100 to 35 m. Finally, the fourth terrace is late Pleistocene in age and occurs between 70 and 25 m.

**Figure 3:**
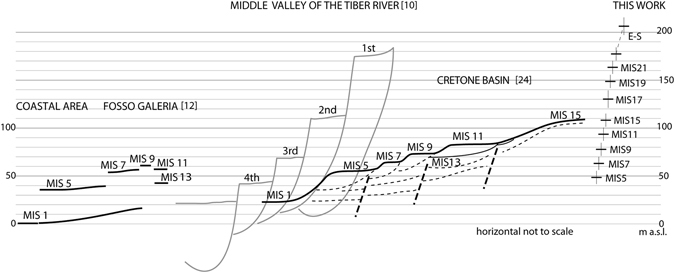
Comparative terrace dataset. Elevation of the terraced surfaces in the coastal area of Rome^12^ and in the Cretone Basin^24^, which are correlated with the MISs through geochronologic constraints, are used as comparative datasets for the paleo-surfaces reconstructed in this work. Comparison with previous work by^10^ is also shown. In comparing these data it must be considered that elevation range for the terraces reconstructed by^9^ is estimated in a much longer portion of the Tiber Valley, accounting for a larger downstream gradient (see text for further comments).

A scheme for the suite of terraces reconstructed in ref. 10 is given in Fig. 3, where the alluvial plain of the Tiber River is reported at 22 m.s.l., in order to compare with the geochronologically constrained geomorphological setting in the Cretone Basin^24^, and that reconstructed in the area investigated in the present work, corresponding to the southernmost portion of the area investigated by^10^.

### Methodological approach

The link between sedimentation and sea-level changes in the area of Rome has been established in the last two decades by a series of studies that, using ^40^Ar/^39^Ar ages of pyroclastic layers intercalated within the sedimentary deposits, have correlated a series of aggradational successions with as many sea-level rises associated with odd-numbered MISs since 870 ka^25–30^. In light of the documented interplay between sedimentary and morphological processes and glacio-eustasy in this region^10^, have identified a series of terraced surfaces in the near-coast valley of the Galeria Stream, interpreted as remnants of alluvial plain surfaces of the Tiber formed during different sea level highstands related to Pleistocene glacio-eustatic oscillations, and successively raised at different elevations by tectonic uplift^31^. Have provided further geochronologic and geometric constraints for these paleo-surfaces allowing^12^ to reconstruct the MIS 9 through MIS 5 terraces along the coast between Civitavecchia and Anzio, and in the area comprising the Tiber and Aniene hydrographic networks, as far as 30 km inland. Similarly^24^, have reconstructed through morpho-structural analysis five terraces within the Cretone lacustrine basin (Fig. 3) and have correlated them with odd-numbered MISs, following the conservative principle of assigning a progressive MIS number starting from the present-day alluvial plain of the Tiber River, which matches MIS 1, and with the exclusion of MIS 3 which is now considered a minor interstadial rather than a highstand, as well as of MIS 13 for the reason explained in the section describing the reconstruction of the paleo-surfaces. The elevations of the MIS-related Cretone terraces are consistent with those that have been correlated, by means of geochronologic constraints, to the different odd MIS’s in the coastal area of Rome (see Fig. 3). In particular, the relative elevations of the terraces within the Cretone Basin are consistent with the occurrence of a late uplift pulse of approximately 45 m since 250 ka, which accounts for the large difference in elevation between MIS 1 and MIS 7, and for the fact the MIS 5 terrace occurs at lower elevation than that of MIS 7, despite the higher sea level reached during this younger isotopic event (Fig. 3).

### Geomorphologic analysis

**Figure 4:**
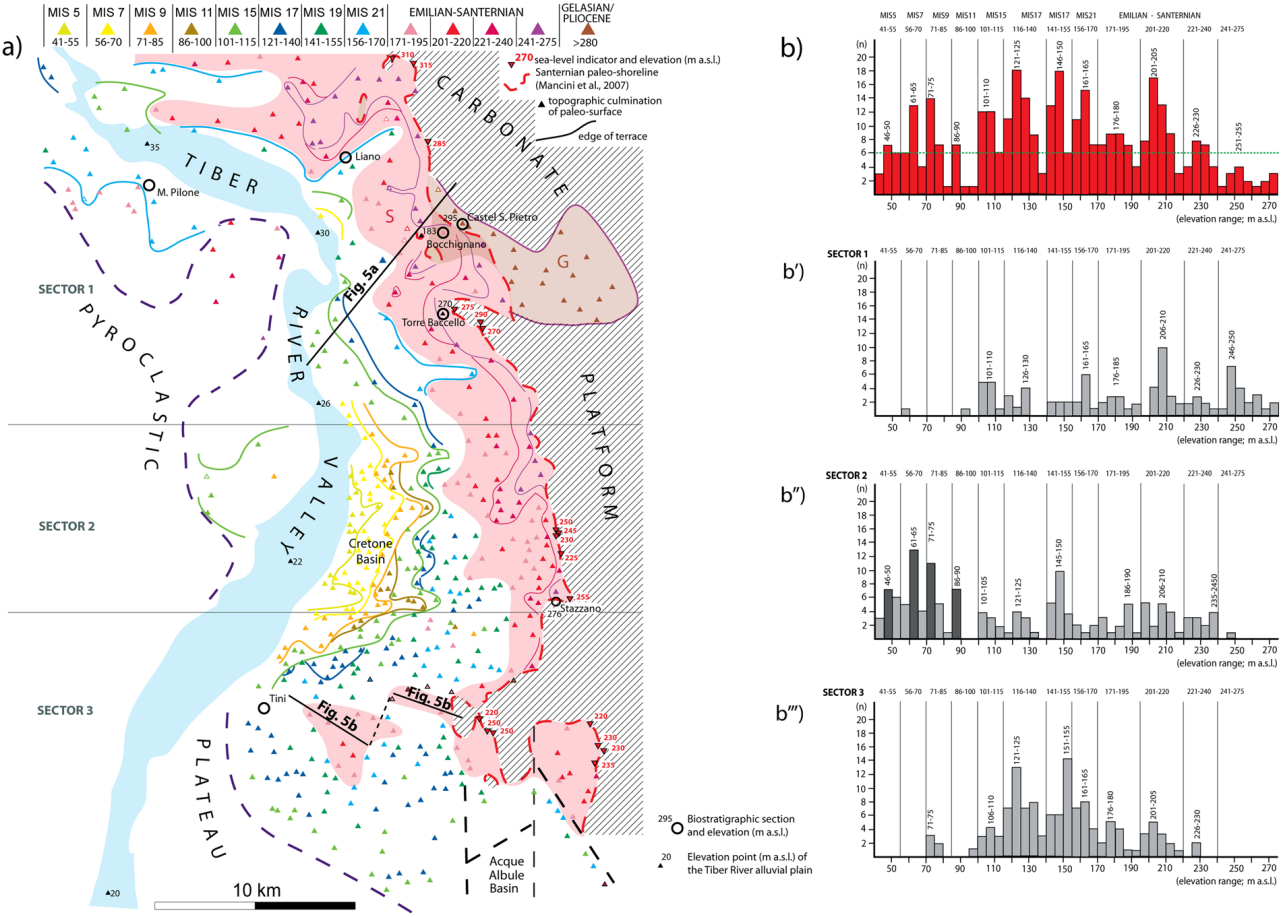
Geomorpholoic map and statistics. Map hand drawn with Adobe© Illustrator CS3 13.0.0 graphic program. (**a**) Geomorphologic map of the Tyrrhenian Sea margin of Latium in the area adjacent to the paleo-shoreline indicators (red triangles with downward vertex) of the Santernian coastline (red dashed line^10^). A series of terraced surfaces is reconstructed following criteria established in ref. 12 and described in the text, from a dataset of topographic culminations (colored triangles with upward vertex); the colored lines are the inner margin of terrace. The red shaded area represents the oldest paleo-surface corresponding to the top of the continental deposits of late Santernian-Emilian age. (**b**) Histograms reporting distribution and peaks of concentration for the topographic culminations, for three different geographic sectors (**b′**–**b″**–**b′′′**) and for the total area (**b**). A series of classes of elevation, identifying the same number of paleo-surfaces are recognized and correlated with the sea-level highstands of marine isotopic stages (MISs), following criteria described in the text. Location of the investigated paleontological sites, the sedimentary sections and the two cross-section of Fig. 5 is shown.

In the present study, terraces along the Tiber River valley have been mapped following the geomorphological approach described in ref. 12), based on the identification of a set of flat surfaces characterized by topographic culminations with elevation ranging through a few meters around a mean value. Selected topographic culminations of the reconstructed terraced surfaces are detected on the 1:25.000 topographic maps of Italy edited by Istituto Geografico Militare (sheets: 144 I SO, 144 I NO, 144 II NO, 144 II SO, 144 III NO, 144 III NE, 144 III SE, 144 IV NO, 144 IV NE, 144 IV SO, 144 IV, SE). They include all the hilltops (i.e. each elevation point within a closed, 5 m spaced isoline) and other quasi-equivalent points within almost closed isolines bordering plateau-like sectors (colored triangles in Fig. 4a). All the topographic elevations used in the analysis are reported in Table 1. Distribution of the hilltop elevations has been statistically analyzed in order to verify the occurrence of discrete elevation intervals corresponding to peaks of concentration, which can be assumed as the mean value for each paleo-surface. Three sectors have been considered as a function of their N-S location along the Tiber Valley (Sector 1–3 in Fig. 4a), and analyzed separately in order to account for downstream gradient.

**Table 1:** Hilltop elevation.

HILLTOPS ELEVATION (m a.s.l.)		
**S 1 (92)**	**S 2 (152)**	**S 3 (134)**
337	247	229
272	237	226
271	237	217
268	237	213
263	237	212
263	231	208
262	231	207
259	228	206
256	226	205
254	226	202
253	223	201
252	221	201
252	221	201
250	217	200
246	214	200
241	214	196
234	212	191
233	211	186
231	211	185
231	207	181
231	206	181
229	206	181
228	206	180
226	206	180
225	202	180
223	198	176
218	197	176
217	197	172
213	196	172
213	196	170
212	193	169
210	187	168
209	187	166
208	187	165
208	187	165
206	186	164
205	182	163
204	182	161
203	177	161
203	172	161
203	171	161
202	171	161
201	168	160
201	167	159
201	163	159
195	157	158
195	156	157
189	152	157
185	152	156
184	151	155
184	151	155
180	149	155
176	149	154
176	149	152
174	149	152
171	148	152
170	148	151
165	147	151
165	147	151
165	146	151
162	146	151
162	143	151
161	142	151
157	142	150
156	141	150
151	141	149
151	131	146
150	127	146
150	126	146
141	126	145
141	124	145
127	124	144
127	123	143
126	121	143
126	116	143
125	115	140
120	111	137
118	106	137
118	106	135
111	106	134
110	105	133
107	104	132
106	103	132
106	103	131
106	89	131
104	88	131
103	87	131
103	87	129
102	87	129
101	86	127
91	86	126
60	86	126
	83	126
	77	126
	76	125
	76	125
	76	125
	76	124
	74	124
	74	122
	74	121
	73	121
	73	121
	72	121
	72	121
	72	121
	72	121
	72	121
	70	120
	68	119
	67	119
	66	117
	66	116
	65	116
	64	116
	63	112
	63	112
	63	111
	63	110
	62	110
	61	107
	61	106
	61	102
	61	101
	61	101
	61	99
	60	77
	60	76
	59	71
	58	71
	57	71
	56	
	56	
	56	
	56	
	56	
	54	
	52	
	52	
	52	
	52	
	51	
	49	
	48	
	47	
	46	
	46	
	46	
	46	
	45	
	44	
	42	

Results of the statistical analysis for the entire area are reported in histogram of Fig. 4b, showing the recurrence (number of data in the y axis) of the elevation of all the hilltop and plateau culminations detected on the 1:25.000 topographic maps. Eleven peaks of elevation values (above a threshold value of n = 6) are recognized in Fig. 4a. An interval ranging 15 to 25 m (bordered by the grey vertical lines) has been arbitrarily assigned as a function of the data clustering, in order to define the corresponding class of elevation pertaining to each paleo-surface. A different color has been assigned to each class of elevation and used to distinguish the topographic points (triangle) of the corresponding paleo-surface in Fig. 4a. Moreover, three geographic sectors have been analyzed separately (Fig. 4b′,b″,b′′′) to account for possible different elevation of the terraced surfaces, due to the hydrographic gradient and to the differential uplift that affected the northern sector with respect to that to the south.

The described procedure allowed us to verify that each one of the classes of elevation values established based on the overall statistics (reported above each column) matches a peak (marked by the corresponding mean range of elevations within the column) in the histograms.

### Biostratigraphic data

The biostratigraphy of the Liano, Tini, Monte Riccio, Monte Pilone and Monte Mario sections provides important bathymetric constraints to the Santernian sedimentary basins in the investigated area (Fig. 1). Following a general regression during the Gelasian (*G. inflata* biozone)^5^, a new ingressive phase affecting the marine basins adjacent to the Apennine margin starts since 2.1 Ma, corresponding to the upper part of the *B. marginata* zone, and the maximum sea level is reached at the beginning of the Santernian, around 1.8 Ma. Circalittoral clay sediments with *B. etnea* and *G. calabra* occur up to 120 and 90 m a.s.l. in Liano and Tini, respectively^32, 33^, whereas an infralittoral to circalittoral environment is associated with the Farneto silts at Monte Mario^34^, reaching up to 125 m a.s.l.^35^.

**Figure 5:**
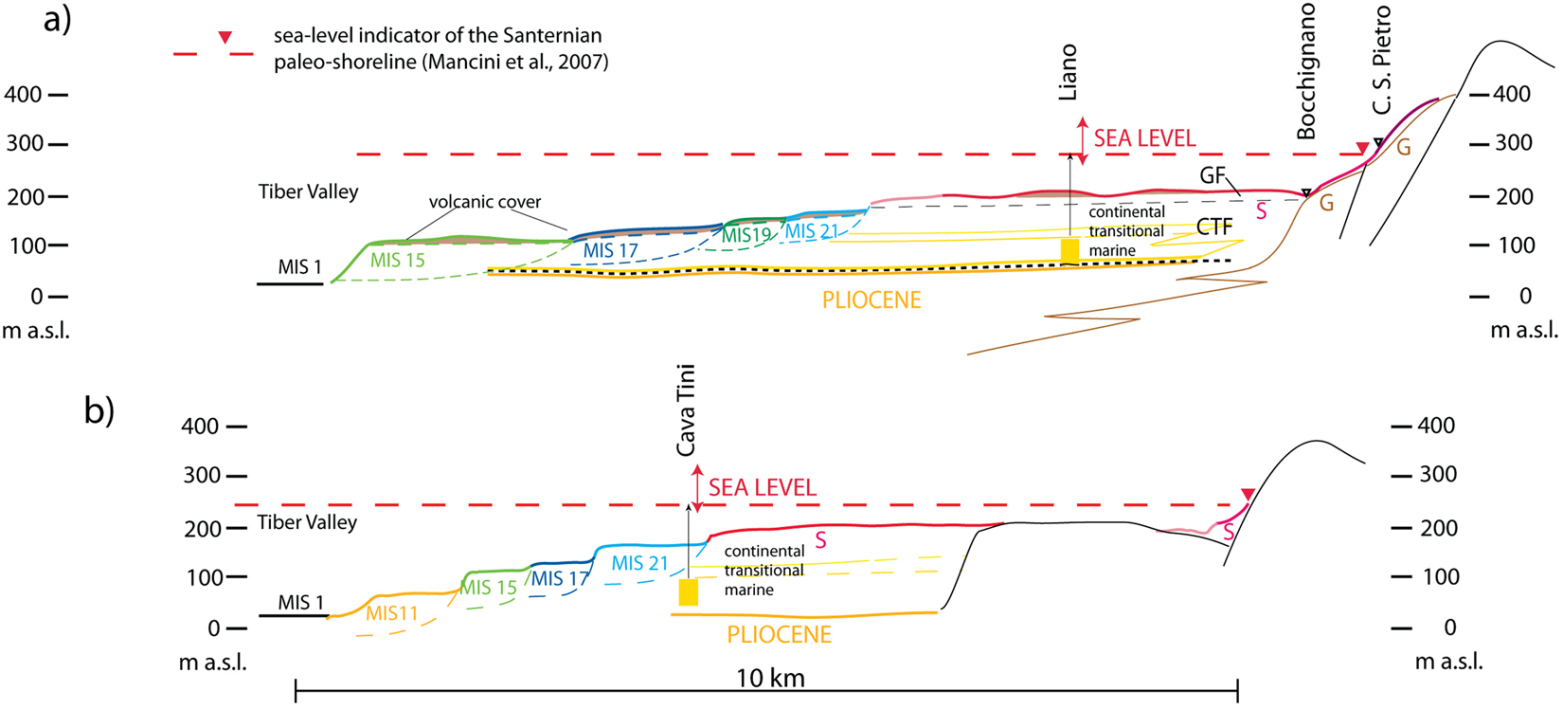
Geologic sections. (**a**,**b**) Schematic cross-sections reconstructing the geomorphologic and stratigraphic setting on the Tyrrhenian Sea margin of Latium (see location in Fig. 4).

Even assuming a conservative estimation for this region for the circalittoral environment at 100–200 m depth (red arrows in Fig. 5), with respect to the canonical range spanning 150–250 m^36^, and at 50–100 m depth for the shallower environment of the Farneto silts, the corresponding altitude intervals account for consistent elevation of the Santernian shoreline (310–270 m a.s.l.) and the corresponding bathymetric feature of the marine basins at this age.

However, a rapid sea-level fall in the order of 120 m (from 270 to ca. 150 m, considering a coastal batymetry ranging 25 m) was achieved in the mid-Santernian (1.7 Ma), as documented by the occurrence of near-shore deposits hosting faunal assemblages of the Tasso Faunal Unit (1.95–1.7 Ma; refs 37 and 38), at an elevation around 125 m a.s.l., either close to the modern coast, (Monte Riccio, ref. 39) and mid-way to the Apennines (Monte Mario, ref. 40) (Fig. 1). In particular, a faunal assemblage referable to the Tasso FU was recovered within a bio-detrital deposit of coastal environment cropping out at 125 m a.s.l. at the Monte Riccio locality, near Tarquinia (Viterbo, Latium)^39^. Similarly, a molar attributed to *Mammuthus meridionalis* ^40^ was recovered from the sands and clays of the Monte Mario Formation referred to the middle Santernian^34^, directly overlying marine silts with *B. etnea* of the Farneto silts^35^. The morphological features of this specimen are similar to those of *M. meridionalis meridionalis*, a form typically diffused in the faunal association from the Middle Villafranchian to the Olivola and Tasso FU^38^. The elevation at which the fossil was found is not recorded, although the Monte Mario sands occur at elevation ranging 100–140 m a.s.l.

Finally, a fragmentary horn of *Bison (Eobison) degiulii* was found within coastal sand deposits^41^ occurring 120 m a.s.l., at the foothill of Monte Soratte, at the Monte Pilone locality. This taxon provides a biochronological indication spanning Pirro to Slivia FUs^42^, and suggests the permanence of a coastline at around 150 m a.s.l. until 1.6 Ma, at the least.

### Reconstruction of the paleo-surfaces

Results of the geomorphological and statistical analyses are summarized in the string at the top of Fig. 4a, showing the correlation between the MISs and the identified paleo-surfaces. Remarkably, statistics for sector 2 confirm the geomorphologic analysis conducted in the Cretone Basin by^24^, accounting for the occurrence of four marked peaks (darker columns in Fig. 4b″) corresponding to the same number of paleo-surfaces ranging 86–90, 71–74, 61–65, and 46–50 m a.s.l. These four terraces are correlated with MIS 11 through MIS 5, along with a less evident, higher one occurring at 101–105 m a.s.l., correlated with MIS 15. According to the less pronounced sea-level rise during MIS 13 with respect to both the previous and the following highstands, the corresponding terrace is buried beneath that of MIS 11, also according to evidence from the Cretone Basin^24^ (Fig. 3); therefore, a paleo-surface correlating with MIS 13 is not expected. When the entire set of paleo-surfaces detected in Fig. 4a is considered, each terrace starting from the lowest one is associated with a progressively older MIS, from MIS 5 to MIS 21, with the exclusion of MIS 13, following the simple geometric criterion and based on the assumption that large glacio-eustatic fluctuations have been associated with the 100 ka cyclicity since MIS 22 (see ref. 11 for a discussion).

While the correlation of the lowest five terraces with MIS 15, MIS 11, MIS 9, MIS 7 and MIS 5 has robust geochronologic and geomorphologic constraints, the proposed correlation with MIS 21 through MIS 17 for the higher paleo-surfaces is a tentative one, lacking chronostratigraphic support. Similarly, we have generically assigned the three paleo-surfaces above 170 m a.s.l., encompassed within the red shaded area in Fig. 4a, to the Santernian-Emilian interval (1.8–1.2 Ma), based on their geometric relationship with the Santernian paleo-shoreline assessed in ref. 10. Although a Gelasian age for this paleo-shoreline has been claimed^16^, results of the present study are consistent with the hypothesis by^10^, as discussed in the next section.

### The Santernian-Emilian paleo-surfaces

A larger paleo-surface ranging 201–220 m a.s.l. is recognized (red triangles in Fig. 4a) and interpreted as the oldest terrace in this area, corresponding to an early coastal plain of late Santernian-Emilian age (S in Figs 4a and 5a,b). This paleo-surface onlaps (“rubine” red and violet contour lines in Fig. 4a) on the older Gelasian continental deposits (G in Figs 4a and 5a) and on the carbonate structure of the Apennines, while it is poorly preserved (pink triangles) closer to the Tiber valley, where a suite of younger terraced surfaces and a related aggradational succession is present. The younger terraces have been correlated with the highstands of the sea level during MIS 21 through MIS 5, as described in the next section.

The cross-section of Fig. 5a shows the composite Santernian-Emilian paleo-surface with the larger, sub-horizontal portion (red line) corresponding to the class of maximum concentration of hilltops ranging 201–205 m a.s.l. (red triangles in Fig. 4a). The portions onlapping on the older Gelasian continental deposits along the Appennine margin (G in cross-section of Fig. 5a) are indicated with the “rubine” red and violet lines, corresponding to the equivalent contour lines in Fig. 4a. Finally, the less preserved, lowest portion of this composite paleo-surface is indicated by the pink line in the cross-sections of Fig. 5a,b.

The Santernian-Emilian continental deposits (S in cross-section of Fig. 5a and b), above which this composite paleo-surface developed, correlate with those of the Chiani-Tevere Formation (CTF) of^10^, with the highest portion corresponding with the Giove Formation (GF). Consistent with its elevation range between 271 and 171 m a.s.l. (Fig. 4b) and the Santernian-Emilian age (1.8 to 1.2 Ma) of the deposits above which it developed, this composite paleo-surface correlates, at least in part, with the highest surface forming a tabular plateau at 250–270 m a.s.l., dated by^10^ to 1.3 Ma. However, it is also apparent that the elevation range of this paleo-surface corresponds to that of the first fluvial terrace recognized by^10^, to which these authors assign an age between 1.2 and 0.6 Ma. In contrast with this interpretation, in the present work we recognize a suite of four paleo-surfaces at lower elevation, ranging from 170 to 100 m a.s.l., that we correlate with odd MISs 21 through 15, dated to 0.86 to 0.6 Ma.

Based on this paleogeographic reconstruction, continental deposits hosting fossil remains of Middle Villafranchian (G in Fig. 5a) to Late Villafranchian age (S in Fig. 5a) are expected to emplace in the interval 2.05–1.7 Ma, corresponding to the Coste San Giacomo, Olivola, and Tasso Faunal Units (FUs)^37, 43^, in the transitional sector between the Apennine chain and the uplifting Santernian coast. During this time span this region was characterized by a foothill landscape in which conglomerate deposits interdigitated with fluvial and lacustrine deposits in fan-delta environments. Consequently, the resulting stratigraphic setting is characterized by frequent vertical and lateral contacts between deposits with ages corresponding to the abovementioned FUs. Therefore, the occurrences of fossil species that do not persist in the Santernian (i.e. limited to the Coste San Giacomo and Olivola FUs) in the continental deposits ranging from 180 to 300 m a.s.l. in the Sabina region (e.g. Bocchignano and Castel San Pietro localities; black triangles in Fig. 5a), cannot be considered as evidence to reject such an age for the paleo-shoreline occurring at 310–270 m a.s.l. in this area.

### Late Santernian regression

A marked regressive phase that would have led to the complete emergence of the Chiani-Tevere basin must have started already during the Santernian, as suggested by the occurrence of *M. meridionalis meridionalis* ^40^ in the sand deposits of Monte Mario, a few meters above the Farneto silts. This taxon did not persist after the Tasso FU (2.05–1.7 Ma)^37^ and its presence within the coastal sediments of Monte Mario implies a rapid sea-level fall in the time span 1.8–1.7 Ma. Similarly, at Monte Riccio the regressive deposits (bio-detrital coarse sand) include a faunal assemblage of the Tasso FU^39^. The coastal deposits of Monte Riccio occur 125 m a.s.l., at the same elevation as similar shallow-water deposits (intercalating gravel layers) that at the Liano section overlie the circalittoral Santernian deposits and, unless there has been significant tectonic dislocation between these two sections, testify to the occurrence of an approximately 120 m sea-level fall (from 270 to ca. 150) by 1.7 Ma.

**Figure 6:**
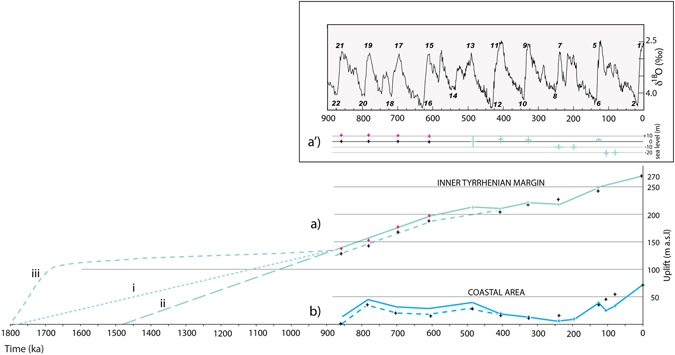
Uplift history. The uplift affecting the investigated area since 900 ka (green solid line) is assessed based on elevation of the terraced surfaces correlated with the MISs (black crosses) corrected for the different sea-level with respect to present day at each interglacial (**a**′; green crosses). The coeval uplift assessed on the Tyrrhenian Sea coast is also shown (blue line; ref. 12). Three different uplift histories are hypothesized for the 1.8–0.9 Ma interval, assuming a constant rate since 1.8 Ma to reach the elevation at MIS 21 (i), assuming the same rate as that characterizing the 0.9–0.5 Ma interval (ii), and considering biostratigraphic data from Monte Mario and Monte Riccio (see text) suggesting a sea-level fall of ca. 120 by 1.7 Ma (iii). Isotopes curve in a′ hand drawn with Adobe© Illustrator CS3 13.0.0 graphic program.

Such a rapid sea-level decline is consistent with a glacio-eustatic fluctuation, rather than with an isostatic uplift. The estimated sea-level fall of ~120 m is in the order of those characterizing the 100 ky cyclicity since 0.9 Ma, while it is higher than the estimation provided for the whole 3.25–1.74 Ma interval in the literature (e.g. ~60 m; ref. 44). However, assessing sea-level changes from paleo-temperature for periods older than 0.5 Ma is a highly speculative procedure. Moreover, the occurrence of the “cold guest” *Arctica islandica* in the Monte Mario sand deposits^45^ was considered as evidence of a strong climatic fluctuation associated with a regressive phase (“Acquatraversa Phase”^46^), supporting the occurrence of a marked sea-level fall triggered by a global cooling. Indeed, combined biostratigraphic and structural data presented in this work suggest that a strong cooling around 1.8 may have caused a dramatic change in the icesheets and a permanent lowering of the average sea-level in the order of ~120 m. Alternatively, we have to assume that an equivalent tectonic uplift occurred in 100 ky, between 1.8 and 1.7 Ma (dashed green c curve in Fig. 6a).

### Fluvial terraces of the Tiber Valley

Inner edges of the terraced surfaces have been indicated in the map of Fig. 4a with the corresponding color in the central-northern area (sector 1 and 2), whereas they are omitted in the southern sector, due to the more complex pattern which is likely the result of poorer preservation, linked with the presence of a more developed hydrographic network. It should also be remarked that the paleo-surfaces that are outlined in Fig. 4 in many instances do not correspond with the top of a sedimentary deposit, since a pyroclastic cover with thickness of up to several meters is discontinuously present. Emplacement of these pyroclastic deposits, mainly during the time span 600–150 ka^22, 47^, modified the original paleo-surfaces by filling paleo-incisions and by mantling the terraces with a variably thick cover. However, the mantling can be considered homogeneous for the paleo-surfaces older than 600 ka, and it does not affect their relative difference in elevation significantly, as shown in the cross-section of Fig. 5a.

In any case, the reconstructed paleo-surfaces should not be considered the top of an aggradational succession, but a terrace resulting from the coupled depositional and erosional processes, which may develop above deposits of different ages. Therefore, they should be used carefully to assign absolute ages to the underlying deposits.

In particular, much caution is required in comparing the terrace reconstruction by^10^ with that performed here. A smaller elevation interval is expected for the terraced surfaces occurring in the area investigated in the present study, with respect to the terraces reconstructed by^10^ in a wider area, encompassing a large upstream portion of the Tiber Valley. Moreover, ref. 10 recognized only four terraces, with respect to the eight reconstructed here in the time span 0.86–0.125 Ma. This fact implies that different paleo-surfaces have been included in the same order of terrace by^10^, also according to the large elevation interval characterizing the terraces reconstructed in their work. As discussed in the previous section, the reconstruction of^10^ identifies a single terrace in the time span 1.2–0.6 Ma, despite the occurrence of four sea-level high-stands (MIS 21 through MIS 15). However, the elevation range of the first fluvial terrace of^10^, ranging from 210 to 170 m a.s.l., corresponds to the composite paleo-surface that in the present study is dated 1.8–1.2 Ma. In contrast, a paleo-surface occurring at 105–100 m a.s.l. is geochronologically constrained and correlated with MIS 15 in the Cretone Basin^24^, and has a wide morphologic representation in the investigated area (Fig. 4), clearly indicating an age older than 0.6 Ma for all the paleo-surfaces occurring at higher elevation. This marked MIS 15 paleo-surface corresponds in part to the second fluvial terrace reported at 210–65 m a.s.l. in ref. 10.

Finally, the two lowest fluvial terraces of^10^, also according to the geochronologic constraints provided by these authors, correspond to those of MIS 7 and MIS 5, which have marked morphological evidence in the whole hydrographic network of the Tiber River, due to the occurrence of the most recent pulse of uplift since 250 ka^12^.

## Discussion

Cumulated terrace elevations and the average elevation of the Santernian coastline in the Sabina area (270 m a.s.l.) are plotted against time in Fig. 6 after correction for the difference in sea level with respect to the corresponding MIS and the present time (Fig. 6a′, see also ref. 12), in order to detect rates of overall uplift during the last 1.8 Ma. In^12^, sea-level elevation during MIS 15 through MIS 21 was assumed equal to Present (black crosses in Fig. 6a), due to the lack of quantitative data in the literature. However, according to general assumptions based on qualitative estimation deriving from the δ^18^O values at corresponding highstands (see ref. 11 for a discussion), in the present study we also propose a solution accounting for an average −10 m elevation for the maximum sea level at MIS 21 through MIS 15 (pink crosses in Fig. 6a′), with respect to MIS 1 (solid portion of the green and blue lines between 0.86 and 0.5 Ma in Fig. 6a,b). Estimates from^48^ (green crosses in Fig. 6a′) are used instead for the younger MISs. Finally, the maximum sea level corresponding to the Santernian shoreline is not easily determinable, and is assumed to be equal to that of MIS 1. This approximation affects the reliability of the estimation of the uplift rate in the time-span 1.8–0.9 Ma, so assumptions made about this period should be considered as speculative.

Assuming that regional uplift has been active since 1.8 Ma, a smaller uplift rate is inferred in the time span 1.8–0.8 Ma (solution i in Fig. 6a) with respect to that estimated in the following period (solid green line), suggesting that the uplift on the inner Tyrrhenian margin during the last 0.8 Ma comprises the combination of the early steady trend (interpretable as the isostatic component) and the trend observed near the coast amended from local tectonic subsidence (interpretable as the magmatic component). Alternatively, steady uplift can be assumed to occur only following 1.5 Ma (solution ii in Fig. 6). Finally, a third possible trend (iii in Fig. 6a) is inferred, considering the rapid sea-level fall of approximately 120 m that occurred in the initial stages of the regressive phase, as suggested by biochronologic indicators at Monte Mario and Monte Pilone. An inferred glacio-eustatic sea-level fall of ca. 120 m is therefore subtracted from the regional uplift (iii dashed line) in Fig. 6a. Stable conditions (with a small isostatic uplift component) are assumed in the interval 1.6–1.1 Ma, after the fast sea-level fall, in order to fit the elevation of the reconstructed paleo-surfaces, and according to the younger age within the Pirro through Slivia FU (1.6–1.1 Ma) for the littoral deposits of Monte Pilone, also occurring around 125 m a.s.l. In this hypothesis, the actual tectonic uplift occurred only after 0.86 ka, coincident with the initial magmatic impulse leading to the start of the volcanic activity of the Roman Magmatic Province around 0.8 Ma^22, 49^. It should be remarked, however, that a change in uplift rates in coincidence with the start of the 100 ky eccentricity-driven climatic cycles at ~0.9 Ma is a worldwide pattern (ref. 50, and references therein), which has been suggested to be related to lower crustal flow triggered by increased erosion brought about by the greater severity of the 100 ky climatic cycles^51^. We note that, rather than being in conflict with the assumption of a major magmatic component for the uplift observed after 0.9 Ma in this region, the rheological mechanism invoked above can be a concurrent cause in triggering magma migration from the lower crust.

A steady uplift rate characterizes the time span 800–600 ka, as inferred from elevation of paleo-surfaces correlated with MIS 21 though MIS 15 in the Tiber Valley. It is followed by a substantially stable trend until 250 ka, and by a new uplift phase until the Present (Fig. 6a). The fact that elevations of the MIS 21 through MIS 15 terraces plot along a rectilinear trend supports the consistency of the assigned ages in the hypothesis that a constant uplift rate characterized this time span. Remarkably, the uplift between 800 and 500 ka parallels the two pulses observed in the coastal area in this time span, amended with reference to the intervening local subsidence. Indeed, this long uplift phase is coincident with the onset of magmatic activity around 800 ka, culminating in the paroxysmal, explosive phases at Colli Albani and Monti Sabatini volcanic districts since 589–561 ka. In contrast, a substantially stable regime corresponds to the large explosive phases of activity 550 through 250 ka in the inland area, consistent with the lack of uplift in the coastal area, where only the subsidence component is observed. Finally, a steady uplift rate resumed inland while it occurred in two steps separated by a tectonic collapse on the coast, during the late hydromagmatic phase at Colli Albani after 200 ka, which is interpreted as a rejuvenated volcanic cycle culminating in the Albano Crater activity, which occurred 70 thorough 36 ka^52^.

## Conclusion

The reconstructed suite of fluvial terraces in the Tiber Valley north of Rome accounts for an overall uplift in the order of 150 m following the mid-Santernian regression, characterized by a rapid and permanent sea-level fall between 1.8 and 1.7 Ma, consistent with global climate indications of a marked cooling at 1.8 Ma, followed by moderate fluctuations, typical of the 41 ky cyclicity, around a substantially stable average value until around 0.9 Ma.

After this time, two pulses of uplift are recognized at 0.86–0.5 Ma, and 0.25 Ma - Present, according to the occurrence of two major volcanic phases in this area^12^, and in agreement with the more plausible hypotheses on the genesis of the Middle Pleistocene high-K magmatism in the Roman Region and the related uplift, which are interpreted as the combined result of slab detachment and metasomatization since around 0.8 Ma^4, 7, 8, 53^.

Consistent with this geodynamic framework, we recognize a major magmatic component, which should be considered responsible for the differential uplift between the northwestern and the southeastern portion of the Santernian paleo-shoreline, as the area of maximum uplift corresponds to the acid intrusive bodies that extend from Tuscany to northern Latium (Elba and Giglio plutons, Tolfa-Cerite structural height; refs 3 and 54, and the vertical displacement progressively decreases southeast. This magmatic component is superimposed on a smaller isostatic component of regional uplift, consistent with the location of the investigated area at the margin of the Apennine orogenic belt, and with he occurrence of major normal faults bordering the Tiber Graben and decoupling the tectonic regime of this sector subjected to crustal thinning, with respect to that occurring in the thickened mountain range domain.
